# A Novel Expedient

**Published:** 1885-08

**Authors:** 


A NOVEL EXPEDIENT.—*The California Medical Journal* tells of a surgeon who, being called to a servant girl who had, in her sleep, swallowed her set of artificial teeth—four canine [?] and two bicuspids, on a vulcanite plate—pushed the plate into the stomach, (not being able to extract it from the aesophagus),and then fed the girl on spool cotton chopped fine, and beat up with white of egg. The plan succeeded, and in due time the foreign body was expelled "without pain or inconvenience," and was found to be wrapped in the mass of thread and egg [?]—so as to round off the corners and prevent injury in its passage. Pretty good yarn.

